# The effectiveness of inpatient rehabilitation after uncomplicated total hip arthroplasty: a propensity score matched cohort

**DOI:** 10.1186/s12891-018-2134-3

**Published:** 2018-07-18

**Authors:** Justine M. Naylor, Andrew Hart, Rajat Mittal, Ian A. Harris, Wei Xuan

**Affiliations:** 1 South Western Sydney Clinical School, University of New South Wales, Liverpool Hospital, Elizabeth Drive, Liverpool, NSW 2170 Australia; 2 South West Sydney Local Health District, Locked Bag 7103, Liverpool, NSW 2170 Australia; 3 Ingham Institute of Applied Medical Research, Westfields Liverpool, PO Box 3151, Liverpool, NSW 2170 Australia

**Keywords:** Arthroplasty, Arthroplasty, Hip, Rehabilitation, Physical therapy

## Abstract

**Background:**

Inpatient rehabilitation is an expensive option following total hip arthroplasty (THA). We aimed to determine if THA patients who receive inpatient rehabilitation report better hip and quality of life scores post-surgery compared to those discharged directly home.

**Methods:**

Prospective, propensity score matched cohort involving 12 private hospitals across five Australian States. Patients undergoing THA secondary to osteoarthritis were included. Those receiving inpatient rehabilitation for reasons other than choice or who experienced significant health events within 90-days post-surgery were excluded. Comparisons were made between those who did and did not receive inpatient rehabilitation for patient-reported hip pain and function (Oxford Hip Score, OHS) and ‘today’ health rating (EuroQol 0–100 scale). Rehabilitation provider charges were also estimated and compared.

**Results:**

Two hundred forty-six patients (123 pairs, mean age 67 (10) yr., 66% female) were matched on 19 covariates for their propensity to receive inpatient rehabilitation. No statistically nor clinically significant between-group differences were observed [OHS median difference (IQR): 0 (− 3, 3), *P* = 0.60; 0 (− 1 to 1), *P* = 0.91, at 90 and 365-days, respectively; EuroQol scale median difference 0 (− 10, 12), *P* = 0.24; 0 (− 10, 10), *P* = 0.49; 5 (− 10, 15), *P* = 0.09, at 35-, 90- and 365-days, respectively]. Median rehabilitation provider charges were 10-fold higher for those who received inpatient rehabilitation [median difference $7582 (5649, 10,249), *P* <  0.001]. Sensitivity analyses corroborated the results of the primary analyses.

**Conclusion:**

Utilization of inpatient rehabilitation pathways following THA appears to be low value healthcare. Sustainability of inpatient rehabilitation models may be enhanced if inpatient rehabilitation is reserved for those most impaired or who have limited social supports.

**Trial registration:**

ClinicalTrials.gov Identifier: NCT01899443.

**Electronic supplementary material:**

The online version of this article (10.1186/s12891-018-2134-3) contains supplementary material, which is available to authorized users.

## Background

Total hip arthroplasty (THA) has been heralded as the ‘operation of the Century’ [1]. Large improvements in mobility and patient-reported outcomes are typically observed with changes far exceeding the small-to-moderate effects seen with non-surgical interventions for arthritis [2–4]. These changes are often experienced in the early subacute phase and improvements are evident regardless of underlying aetiology [3, 5, 6]. Such is the success of the operation, annual increases in the volume of surgery in Australia – as it has elsewhere - have been recorded by the Australian Orthopaedic Association National Joint Replacement Registry (AOANJRR) since 2003 [7]. Presently, over 36,000 THA procedures are undertaken annually in Australia with the majority (~ 60%) performed in the private sector [7]. In 2016, 87,733 primary THA procedures were performed in the United Kingdom (UK) [8]. In the United States (US), it is estimated that up to 562,000 THA cases may be undertaken annually by 2030 [9].

Following surgery, physiotherapy-led exercise-based rehabilitation is often prescribed [10]. Evidence in support of such prescription is apparent when physiotherapy-led programs are compared to no or minimal intervention following THA. A recent systematic review of randomized trials concluded that physiotherapy-led exercise programs yielded better gait-related outcomes post-THA when compared to no intervention [10]. Interestingly, consistent with the evidence concerning the more intensive clinic-based rehabilitation programs following total knee arthroplasty (TKA) [11–15], clinic-based one-to-one or group-based programs have not been shown to be superior over monitored or unmonitored home-based programs for the recovery of mobility, function and health-related quality of life after THA [10, 16, 17]. Further, the evidence-base lacks randomized trials comparing inpatient-based rehabilitation with less intensive programs. This gap is important to address as utilization of inpatient rehabilitation is common and it is a comparatively costly pathway [18–20]. Recent Australian data from the private sector indicate the median referral rate per surgeon to inpatient rehabilitation is 29% (range 0–100%) [21]. Inpatient utilization rates vary internationally [22, 23], with reported rates as high as 81% (Japan) and 53% (US) and as low as 0% (Spain, Turkey) and 3% (UK) [24].

Only one randomized controlled trial (RCT) has been published aimed at determining the efficacy of inpatient therapy over a non-inpatient alternative following THA [25]. Eighteen days of inpatient rehabilitation was not more effective nor cost-effective compared to eigth domiciliary (physiotherapy) visits following THA or TKA (combined cohort).

Randomized trials are difficult to undertake in general, but they are particularly difficult to perform in the private healthcare sector where consumer and provider preferences are strong drivers of care delivery [26, 27]. This may partly explain why much of the evidence in this space - showing no superiority of the inpatient pathway - is predominantly provided by retrospective and prospective observational studies and administrative datasets [19, 28–30].

In light of the difficulty in performing an RCT, this study, using a propensity score (PS) matched cohort, aimed to determine whether privately insured THA patients who received inpatient rehabilitation, compared to those who did not, had better patient-reported joint and health scores across the first 12 months post-surgery. Informed by the high- and low-level evidence in this area, we hypothesized that the inpatient pathway would not procure superior patient-reported recovery to a level that was clinically meaningful over the alternative.

## Methods

This study was nested within a national, prospective observational study involving 19 hospitals across five States [31] (ClinicalTrials.gov Identifier: NCT01899443). We have previously reported the results of a related PS matched cohort aimed at determining the effectiveness of a rehabilitation pathway involving inpatient rehabilitation compared to one not involving inpatient rehabilitation following TKA [32]. The PS is the probability of treatment assignment conditional on observed baseline characteristics [33–38]. Here, matching people on their probability of being allocated to inpatient therapy addresses treatment selection bias based on known patient characteristics.

### Ethical approval and funding source

Ethical approval was provided by multiple ethics committees (see *Declarations*). Informed participant consent was obtained. The HCF Research Foundation funded the study, but had no role in study design or data analysis.

### Sampling and hospital recruitment

Hospitals contributing to the AOANJRR [7] performing over 275 TKA or THA procedures in 2012, were eligible to participate in the larger study. Related publications describe the sampling procedure which procured 10 public and nine private hospitals [31, 32, 39].

### Patient enrolment and eligibility

Patient enrolment and eligibility were identical to that described in an aforementioned study [32]. People awaiting arthroplasty secondary to osteoarthritis between August 2013–December 2014 were screened by site coordinators during their pre-admission review. Eligibility criteria for the larger study included primary surgery, no further arthroplasty planned within 3-months, and the ability to understand the protocol and be followed-up by telephone for 1 year. For this nested study, being privately insured was an additional inclusion criterion. Publicly insured patients were explicitly excluded because the few referred to inpatient rehabilitation (20/317, 6.3%) were primarily referred based on a clinical or social need (14/20). To reduce treatment selection bias and improve generalizability to uncomplicated THA, we also subsequently excluded the privately insured patients referred to inpatient rehabilitation due to slow progress, inadequate social supports (e.g living alone or without the help of an able carer), and those with conditions that would alter their rehabilitation pathway (e.g bilateral surgery or significant complication (defined below) up to 90-days post-surgery).

### Data collection

Patient-level variables collected prior to surgery (baseline) included weight, height, insurance status, comorbid conditions including requirement for daily medication, education level, presence of other lower limb or back conditions impairing mobility and patient-reported outcomes measures (PROM). Additional patient-specific data via clinician-completed forms were collected by site coordinators - American Society of Anesthesiologists (ASA) scores, complications, length of stay (LOS), discharge destination, and reason for referral to inpatient rehabilitation if relevant. Patient-reported outcomes, including surveys, ongoing rehabilitation details, complication, and time-off-work (patient, carer) were obtained by researchers by telephone at 35, 90 and 365-days post-surgery. For quality control, clinician-collected data were re-abstracted from the medical record by researchers; significant patient-reported complications were verified by treating hospitals, surgeons or general practitioners.

#### Complications

Acute complications were stratified according to the Clavien-Dindo Classification whereby the code applied (1–5) is based on the type of care deviation necessitated by the complication [40]. People experiencing the more severe complications (coded ≥3) (e.g pulmonary emboli or dislocation), were excluded. Those experiencing a less severe complication, but one potentially affecting longer-term recovery (e.g neuropraxia) were also excluded. Patients experiencing any surgery-related complication requiring re-admission or any unrelated health event requiring hospital admission up to 90 days post-surgery, were also excluded.

#### Outcomes

The Oxford Hip Score (OHS) [41] and the Euroqol Visual Analogue Scale for health ‘today’ (EQVAS) [42, 43], were the primary outcomes. The EQVAS was obtained using a VAS at baseline and verbally at the time of follow-up as the methods have been shown to be equivalent in people awaiting arthroplasty [42].

Rehabilitation charges were determined from the perspective of the provider and the methodology is described elsewhere [32]. Table 1 summarizes the unit costs (AUD) applied for each rehabilitation ‘type’ encountered. Total provider charges for each participant were calculated based on the length of stay in a rehabilitation facility (where applicable), and the type and frequency of community-based therapy received up to 10-weeks post-surgery, including clinic, domiciliary, supervised hydrotherapy, and day hospital visits. Individuals in the ‘No inpatient rehabilitation’ group who also had no community-based rehabilitation were assigned $0 rehabilitation provider charges.

**Table 1:** Unit charges assigned to each rehabilitation ‘type’ encountered

Rehabilitation ‘type’	Unit charge	Comments
Inpatient rehabilitation, per night	$700	Based on 2015 private health insurance reimbursement figures. Note: The Australian Public Hospital Cost Report 2013 [46] cites $967.29 per night government. The figure used in this study was considered conservative.
Day Hospital, per visit	$255.15	Based on The Australian Public Hospital Cost Report 2013 [46] (clinic code 40.12, pg 112).
Private physiotherapy clinic visit	$74.90	Based on COMCARE 2015 [47] rates for a standard consultation or return visit. Same charge was applied regardless of group-based on one-to-one based care.
Domiciliary (hospital in the home) visit	$87.10	COMCARE 2015 [47] rate for a standard visit was used.
Physiotherapy supervised hydrotherapy visit	$74.90	Based on COMCARE 2015 [47] rates. Same charge was applied regardless of group-based or one-to-one based care.

Key: All charges in Australian dollars

### Analysis

The projected sample size for the larger study was ~ 2000. Assuming our recruitment pattern was consistent with patterns observed in the AOANJRR [7], we anticipated the definitive sample would comprise 40% THA recipients (*n* = 800), and most of those (60%) (*n* = 480) would be private insured. We conservatively predicted, therefore, our sample for this nested study would exceed 128 people (64 in each treatment group) which is what would be required (80% power, 0.05 significance level) to detect a minimal important between-group difference of 5 points (0.55 of the baseline SD) [41] in the OHS. A sample of 17 would be sufficient to detect a minimally clinically important improvement of 23 (1.36 SD [42, 43] baseline mean) for the EQVAS.

We assessed normality of the data prior to analysis. Mean, median and percentages were used to characterize the cohorts. Covariates of the unmatched cohorts were assessed using independent t-tests and χ^2^, whilst covariates and outcomes in the matched (paired) cohorts were assessed using paired t-tests, Wilcoxon signed-rank tests and McNemar tests [33, 34].

Propensity score determination and subsequent matching were determined as follows:

i) PSs for the prediction of referral to inpatient rehabilitation were estimated for all eligible patients using unconditional logistic regression [33–35]. Here, the outcome was treatment group and model covariates included variables thought to influence treatment assignment prior to surgery or the primary outcomes [33–35]. Covariates included baseline Oxford and EQVAS scores, age, body mass index (weight [kg]/height [m]^2^), gender, ASA scores, comorbidity (any condition of the cardiovascular, renal, respiratory, endocrine, liver, central and peripheral nervous systems, and excluding musculoskeletal conditions), other lower limb or back problems limiting mobility, surgical approach (‘anterior’ or ‘other’), and education level (Yr 8 or below, Yrs 9–10, Yrs 11–12, Degree qualified). We also included any measured variable that was found to be significantly different between the treatment groups prior to matching. For this reason, employment status was also used for matching.

ii) We assessed the distribution of the PSs across the two treatment groups using visual inspection and descriptive statistics to ensure there was sufficient overlap (area of common support [33–35]) in probabilities to receive inpatient rehabilitation.

iii) Using caliper matching without replacement [32, 33] we then used the PSs to match each patient who received inpatient rehabilitation in a 1:1 ratio with one who did not. A caliper distance of an absolute difference in PS of 0.05 was employed, equating to 0.28 of the pooled SD of the PSs for the unmatched cohorts. Successful matching was indicated by achieving a standardized mean difference (SMD) [34] between the groups < 0.20 for any given variable (differences < 0.1 are considered negligible [36]; differences up to 0.25 are considered tolerable [35]), and an absence of statistical significant difference (*p* <  0.05).

Variables not included in the matching were ‘hospital’ and ‘surgeon’. The inclusion of these caused non-convergence of the estimate in the PS calculation owing to high correlation between surgeon or hospital and treatment group. To assess the influence of ‘hospital’ or ‘surgeon’ on primary outcomes, we performed multivariable linear regression analyses incorporating these variables.

As treatment allocation was not random, we considered the experience of a second arthroplasty (different joint) or later joint-related complication after 90 days as a potential between-group confounder of the primary outcomes. Thus, we conducted sensitivity analyses on the 365-day outcomes excluding persons who experienced such events.

We also performed sensitivity analysis using Rosenbaum bounds [37, 38] on the matched groups to determine the possible effect of unmeasured confounders on the conclusiveness of our results. A sensitivity parameter ‘Gamma’ was defined in the Rosenbaum bounds analysis. The Gamma indirectly informs how large the effect of the unmeasured confounder would need to be to change the conclusiveness of the matched results. The larger the gamma the more robust the result to an unobserved confounder. A Gamma of 1.2, for example, indicates that an unmeasured confounder would need to increase the likelihood of a patient being assigned to the inpatient group by a factor of 1.2 before the conclusiveness of the results are threatened [36, 44].

All analyses were conducted using SAS V9.4 (SAS Institute Inc. Cary, NC, USA) and the psens function in the rbounds package of R. No imputation was undertaken for missing data.

## Results

**Fig. 1:**
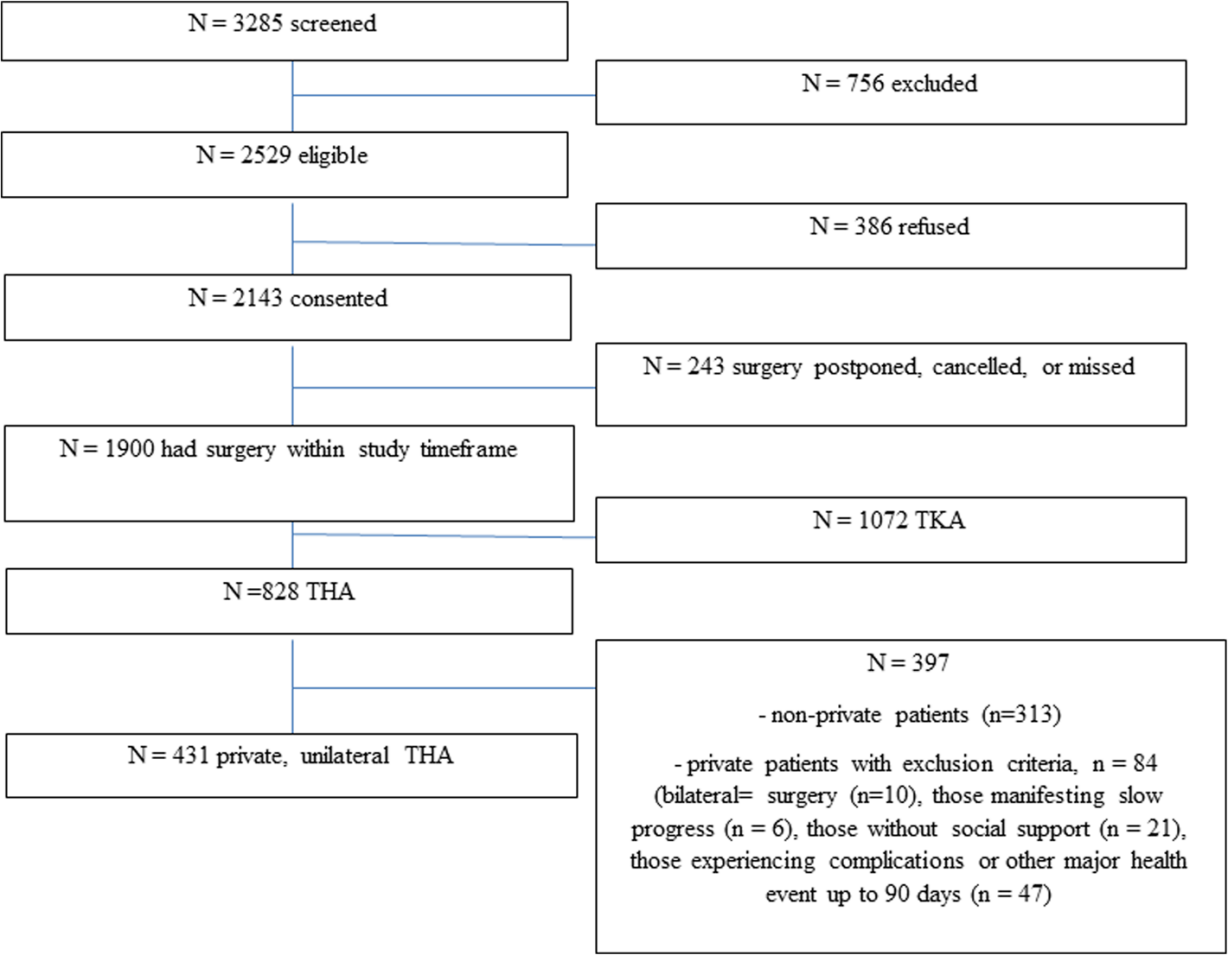
Cohort ascertainment

Figure 1 summarizes cohort ascertainment. From an eligible sample of 431 privately insured primary, unilateral THA patients from 12 hospitals, 123 pairs were matched.

### Unmatched cohort

In the unmatched cohort, a PS was estimable for 403 patients. The mean (SD) propensity score for the sample was 0.34 (0.18). Assessment of the overlap of the PSs across the unmatched groups revealed sufficient common support between the two treatment groups [Inpatient Group, *n* = 136 - mean 0.44 (0.18), min 0.06, max 0.85; Non-inpatient Group, *n* = 267 – mean 0.29 (0.16), min 0.02, max 0.85].

### Unmatched versus matched cohorts

In the unmatched cohort, the inpatient rehabilitation group was older, had more males, had higher levels of comorbidity, and worse baseline OHSs (Table 2). Unadjusted comparisons between the two groups revealed significantly worse health scores at every time point and worse OHS at 90 days for the Inpatient Group (Additional file 1). Matching lowered the SMD for all but one covariate (it went from 0.10 to 0.11); the largest SMD was 0.14 (Table 3). Acute-care LOS was significantly longer in the Inpatient Group in both the unmatched (6.2 (2.2) vs 4.6 (1.7) days) and matched (5.9 (2.1) vs 5.0 (2.0) days) conditions.

**Table 2:** Characteristics of total hip arthroplasty recipients: Pre-matching

	Inpatient Rehabilitation Group, *n* = 141	No Inpatient Rehabilitation Group, *n* = 290	Standardized Mean Difference	*P*-value
Age, mean (SD), y	68.7 (9.9)	63.7 (10.4)	0.50	< 0.001
Male, % (n)	68.1 (96)	47.6 (138)	0.42	< 0.001
BMI, mean (SD)	27.7 (4.9)	28.2 (4.9)	0.11	0.27
Education, % (n)				
1	1.4 (2)	1.4 (4)	0.0	0.97
2	21.3 (30)	18.3 (53)	0.08	0.46
3	56.7 (80)	51.4 (149)	0.11	0.30
4	20.6 (29)	29.0 (84)	0.20	0.06
ASA, % (n)				
1	9.2 (13)	19.1 (55)	0.29	0.009
2	54.6 (77)	62.2 (180)	0.15	0.14
3	33.3 (47)	17.4 (50)	0.37	< 0.001
4	2.8 (4)	1.4 (4)	0.10	0.30
Comorbidity, % (n)				
Nil	8.5 (12)	21.7 (63)	0.38	< 0.001
Yes, but no daily medication	5.0 (7)	11.4 (33)	0.24	0.03
Yes, with daily medication	86.5 (122)	66.9 (194)	0.48	< 0.001
Oxford Hip Score, mean (SD)	22.3 (9.2)	24.4 (8.6)	0.24	0.02
EuroQol VAS, mean (SD)	71.4 (19.0)	73.5 (16.3)	0.12	0.24
Other lower limb or back problems limiting mobility, % (n)	51.1 (72)	42.1 (122)	0.18	0.08
Anterior surgical approach	33.8 (48)	31.0 (90)	0.06	0.56
Paid employment, % (n)	31.2 (44)	44.1 (128)	0.27	0.01

*Abbreviations: BMI* body mass index, *ASA* American Society of Anesthesiology, Education 1 = nil formal; 2 = Year 10 or less; 3 = Year 11 or 12; 4 = Degree or greater; Comorbidity included any conditions of the cardiovascular, renal, respiratory, endocrine, liver, central and peripheral nervous systems, and excluding other musculoskeletal conditions. Note: Standardized mean difference was calculated using the following equation - Standardized Mean Difference^34^ = (Mean1 – Mean 2)/√((SD1)^2^ + (SD2)^2^)/2

**Table 3:** Characteristics of total hip arthroplasty recipients: Post-matching

	Inpatient Rehabilitation Group, *n* = 123	No Inpatient Rehabilitation Group, *n* = 123	Standardized Mean Difference	*P*-value
Age, mean (SD), y	67.8 (10.0)	66.9 (10.6)	0.09	0.48
Male, % (n)	64.2	68.3	0.09	0.50
BMI, mean (SD)	27.8 (4.8)	28.0 (5.1)	0.04	0.76
Education, % (n)				
1	1.6 (2)	1.6 (2)	0	1.0
2	20.3 (25)	22.80 (28)	0.06	0.64
3	55.3 (68)	51.2 (63)	0.08	0.52
4	22.8 (28)	24.4 (30)	0.04	0.76
ASA, % (n)				
1	10.6 (13)	7.3 (9)	0.11	0.37
2	59.4 (73)	61.0 (75)	0.03	0.79
3	28.5 (35)	28.5 (35)	0.0	1.0
4	1.6 (2)	3.3 (4)	0.11	0.70
Comorbidity, % (n)				
Nil	8.9 (11)	8.9 (11)	0	1.0
Yes, but no daily medication	4.9 (6)	3.3 (4)	0.08	0.52
Yes, with daily medication	86.2 (106)	87.8 (108)	0.05	0.71
Oxford Hip Score, mean (SD)	23.1 (8.9)	21.9 (8.2)	0.14	0.27
EuroQol VAS, mean (SD)	71.7 (19.4)	70.8 (16.8)	0.05	0.69
Other lower limb or back problems limiting mobility, % (n)	47.2 (58)	48.8 (60)	0.03	0.80
Anterior surgical approach	33.8 (42)	31.0 (38)	0.06	0.56
Paid employment, No. (%)	34.2 (42)	31.7 (39)	0.05	0.68

*Abbreviations: BMI* body mass index, *ASA* American Society of Anesthesiology; Education 1 = nil formal; 2 = Year 10 or less; 3 = Year 11 or 12; 4 = Degree or greater; Comorbidity included any conditions of the cardiovascular, renal, respiratory, endocrine, liver, central and peripheral nervous systems, and excluding other musculoskeletal conditions Note: Standardized mean difference was calculated using the following equation - Standardized Mean Difference^34^ = (Mean1 – Mean 2)/√((SD1)^2^ + (SD2)^2^)/2

### Outcomes – Matched cohort only

*35- and 90-days:* Table 4 summarizes the comparisons between the matched groups. The Inpatient Group did not have superior outcomes at 35- or 90-days. Time-off-work outcomes were not significantly different. Median rehabilitation provider charges were significantly higher in the Inpatient Group regardless of whether inpatient provider charges were included in the calculation. The Inpatient Group received a greater number of physiotherapy sessions in the community (day hospital as well as clinic) [median difference 5 (IQR 9,0); Inpatient Group - 6 (IQR 2,10), Non-Inpatient Group 0 (IQR 0,3)] (Table 5). A greater proportion of the Inpatient Group were receiving community-based rehabilitation beyond week 10 (23.9% [95%CI 17.1–32.4] vs 12.2% [7.2–20.2]).

**Table 4:** Outcomes at 35, 90 and 365 days in the matched cohorts

	35 days				90 days				365 days			
	Inpatient	No Inpatient	*P*-value	Median difference	Inpatient	No Inpatient	*P*-value	Median difference	Inpatient	No Inpatient	*P*-value	Median difference
OHS median (IQR)					46 (41, 48	46 (41, 48)	0.60	0 (−3, 3)	48 (46, 48)	48 (46, 48)	0.91	0 (−1, 1)
EQVAS today, median (IQR)	80 (75, 90)	85 (80, 90)	0.24	0 (−10, 12)	85 (80, 95)	90 (80, 95)	0.49	0 (−10, 10)	85 (75, 95)	90 (80, 95)	0.09	5 (−10, 15)
Rehabilitation costs, $, median (IQR)					7620 (5796, 10,399)	0 (0, 225)	< 0.001	-$7582 (−10,249, −5649)				
Rehabilitation costs, $, excluding inpatient costs					$824 (87, 2041)	0 (0,225)	< 0.001	-$749 (− 1786, 0)				
Time off paid employment > 6 weeks, % (95% CI)					24 (17, 32)	21 (15, 30)	0.66	-2 (−13, 8)				
Carer time off paid employment, Yes/No, % (95%CI)	13 (8, 21)	13 (8, 21)	1.0	0 (−8, 8)								

Legend: *OHS* Oxford Hip Score, *EQVAS* EuroQol Visual analogue scale

**Table 5:** Profile of community-based supervised rehabilitation received amongst those who received it

	Inpatient Group			No Inpatient Group		
	%	No. of visits, mean (sd)	Median	%	No. of visits, mean (sd)	Median
Receiving community rehabilitation	79	8.7 (4.5)	8	35	5 .4 (3.7)	5
Type of community-based rehabilitation						
1-to-1 clinic	36	5.6 (4.4)	5	37	3.7 (2.4)	3
1-to-1 hydrotherapy	11	6.2 (3.2)	7	11	3.3 (1.3)	3
Group-based clinic	13	6.3 (2.9)	5	11	5.0 (3.6)	6
Group-based hydrotherapy	6	5.3 (3.8)	4	9	4.5 (2.6)	4
Domiciliary	1	1	1	40	6.2 (3.6)	7
Day Hospital program	62	8.7 (3.8)	8	6	7.0 (1.4)	7

Note: The data summarise attendance of those who had ongoing supervised rehabilitation after the acute hospital. Those who did not have ongoing supervised rehabilitation after discharge from hospital are excluded from these calculations. Interpretation example – 62% of those who had ongoing supervised rehabilitation after discharge from inpatient rehabilitation participated in a Day Hospital program compared to 6% of those who did not participate in inpatient rehabilitation. The percentages under ‘type’ are not additive as each type is not mutually exclusive

*365-days:* The Inpatient Group did not have superior outcomes at 365 days; the median OHS in both groups was the maximum score possible (48). Sensitivity analyses for the 365-day outcomes involving 114 pairs, yielded the same results (Additional file 2). In the analyses incorporating hospital and surgeon, neither significantly influenced the primary outcomes at any point (Additional file 3). Sensitivity analysis exploring the Rosenbaum bounds for the possible effect of unmeasured confounders yielded gamma values from 1.1 to 6.0 (Additional file 4).

## Discussion

Results from a related PS matched cohort involving uncomplicated TKA recipients indicated that the inpatient rehabilitation pathway, compared to the non-inpatient pathway, did not provide superior patient-reported outcomes up to 1-year post-surgery [32]. The current study suggests the same is true for THA recipients. Importantly, the median between-group differences for the OHS and EQVAS were much smaller than the differences estimated to be clinically important. Further, the inpatient rehabilitation pathway was associated with far higher rehabilitation provider charges, and similar time-off -work outcomes for patients and carers. Overall, our findings align with the one aforementioned RCT conducted in this area involving hip arthroplasty [25], and highlight the impressive levels of patient-reported recovery typically seen after this surgery regardless of the rehabilitation pathway taken.

Our study has several strengths. The use of propensity-based scoring has allowed us to report the comparison between two rehabilitation pathways in simple, absolute terms of the outcomes whilst keeping measured covariates balanced between the groups. Most importantly, acceptable matching across all our measured covariates was achieved indicating that any differences in the outcomes observed are not attributable to confounding resulting from differences in these variables. Further, we removed other sources of ‘noise’ through our strict eligibility criteria, thus, between-group differences in the rate of complications or other major health events are not a source of bias. Finally, our cohort was derived nationally, thus, the characteristics of the unmatched and matched samples should reasonably reflect private patients who undergo THA in Australia.

Despite its advantages, a limitation of the PS method is that bias from unmeasured covariates that may be influencing treatment allocation or outcomes is not eliminated [33–37]. Here, surgeons and hospitals could be viewed as possible confounders. Our supplementary regression analyses, however, allowed us to adjust for the effect of these with neither having a significant effect on outcomes. We did not match on prosthesis type (as there were too many), though we did adjust for what may be considered more important (surgical approach). There is still a possibility that we have not accounted for all unknown confounders, but we think it unlikely we missed important ones. Our sensitivity analyses on the possible effect of unmeasured confounders indicate that an unknown confounder would need to change the probability of being allocated to inpatient rehabilitation by at least a factor of 1.3 for five of our outcomes in order to change the statistical significance of the results. Further, if an unmeasured confounder changed a result from non-significant to significant, the effect could still favor discharge directly home. Given we have included many of the known potential confounders in our PS analysis and supplemented this with multiple regressions including ‘surgeon’ and ‘hospital’, we contend the important variables are covered. Further, we are not aware of any other unmeasured confounder that the literature points to that would lead us to change our conclusions. That our results concord with the one RCT conducted in this area [25] and several observational studies [28–30], it would appear we have not overlooked major confounding influences.

A potential limitation of our study is the lack of physical performance measures [45]. It remains to be seen if inpatient rehabilitation improves gait or strength more so than rehabilitation provided in other settings. We note gait outcomes have not been shown to be superior in supervised community-based settings compared to home programs when conducted within 8-weeks of surgery [10, 17]. It is possible the same is true for inpatient rehabilitation.

An important caveat is that our results apply to those who have had an uncomplicated recovery and to those for whom discharge destination was a choice. We contend then that the inpatient rehabilitation pathway remains an important option for those with complications, limited social supports, and poor progress acutely. Based on the criteria used here, we estimate that a small minority (approximately 14%) of privately insured patients may require inpatient rehabilitation based on these three conditions (9.1, 4.1 and 1.2% respectively) (Fig. 1). We note, however, recent data from the US suggest discharge to inpatient rehabilitation facilities following THA is associated with increased morbidity [23]. A reasonable approach, therefore, would be to avoid referral to inpatient facilities unless alternatives are exhausted.

A final important caveat is that our cost data pertain only to the first 90 days after surgery and only include rehabilitation provider charges. It is likely that there were continued cost differences beyond this time given a greater proportion of the Inpatient Group were still receiving community-based therapy beyond 10 weeks.

## Conclusions

In conclusion, in the absence of sufficient high-level evidence to date, our study provides robust and useful signals concerning the comparative effectiveness and provider charges of a pathway that includes inpatient rehabilitation after uncomplicated THA. Consistent with the one published RCT in this space [25], our data indicate a rehabilitation pathway incorporating inpatient rehabilitation is not likely to be cost-effective for uncomplicated THA recipients. Our data and data elsewhere support the use of community-based and home-based programs to secure impressive levels of patient-reported recovery.

## Additional files

Additional file 1: Unadjusted analyses. Unadjusted analysis between treatment group and outcome. (DOCX 20 kb)

Additional file 2: Sensitivity analysis 1. Sensitivity analysis for 365 day outcomes. (DOCX 19 kb)

Additional file 3: Sensitivity analysis 2. Sensitivity analysis to determine effect of ‘hospital’ and ‘surgeon’. (DOCX 39 kb)

Additional file 4: Sensitivity analysis 3. Sensitivity analysis to determine effect of the unmeasured confounders. (DOCX 21 kb)

## Abbreviations

95%CI: 95% confidence interval
AOANJRR: Australian Orthopaedic Association National Joint Replacement Registry
ASA: American Society of Anesthesiologists
AUD: Australian dollars
EQVAS: EuroQol Visual Analogue Scale
LOS: Length of stay
OHS: Oxford Hip Score
PS: Propensity score
RCT: Randomized controlled trial
SD: Standard deviation
SMD: Standardized mean difference
THA: Total hip arthroplasty
TKA: Total knee arthroplasty
UK: United Kingdom
US: United States
yr.: Year
