# Supplementary material for: The effectiveness of inpatient rehabilitation after uncomplicated total hip arthroplasty: a propensity score matched cohort
Source: BMC Musculoskelet Disord. 2018 Jul 18;19:236. doi: 10.1186/s12891-018-2134-3 (PMC6052669; doi:10.1186/s12891-018-2134-3)
Supplement: Supplementary file 1 — Unadjusted analyses. Unadjusted analysis between treatment group and outcome. (DOCX 20 kb) [file 12891_2018_2134_MOESM1_ESM.docx]

**Additional file 1**

**The effectiveness of inpatient rehabilitation after uncomplicated total hip arthroplasty: a propensity score matched cohort**

**Naylor JM, Hart A, Mittal R, Harris IA, Xuan W**

**Unadjusted analyses between treatment group and outcome**

| Model | Variable | Beta coefficient | P-value |
| --- | --- | --- | --- |
| EQVAS 35 days | Inpatient Yes/No | -3.5 | 0.0006* |
| EQVAS 90 days | Inpatient Yes/No | -2.54 | 0.049* |
| EQVAS 365 days | Inpatient Yes/No | -3.95 | 0.0027* |
| Oxford Knee Score 90 days | Inpatient Yes/No | -1.57 | 0.006* |
| Oxford Knee Score 365 days | Inpatient Yes/No | -0.82 | 0.077 |

*Inpatient allocation associated with a significantly poorer outcome.
