# Supplementary material for: The effectiveness of inpatient rehabilitation after uncomplicated total hip arthroplasty: a propensity score matched cohort
Source: BMC Musculoskelet Disord. 2018 Jul 18;19:236. doi: 10.1186/s12891-018-2134-3 (PMC6052669; doi:10.1186/s12891-018-2134-3)
Supplement: Supplementary file 2 — Sensitivity analysis 1. Sensitivity analysis for 365 day outcomes. (DOCX 19 kb) [file 12891_2018_2134_MOESM2_ESM.docx]

**Additional file 2**

**The effectiveness of inpatient rehabilitation after uncomplicated total hip arthroplasty: a propensity score matched cohort**

**Naylor JM, Hart A, Mittal R, Harris IA, Xuan W**

**Sensitivity analysis for 365 day outcomes**

**Excluding those who had a second arthroplasty (other joint) (n = 7) or other major health event* (n = 2) after 90 days**

|  | Inpatient Group, n = 114 | No-inpatient Group , n = 114 | Median difference (IQR) No Inpatient minus Inpatient | P-value |
| --- | --- | --- | --- | --- |
| Oxford knee score, median (IQR) | 48 (46,48) | 48 (46, 48) | 0 (-1, 1) | 0.89 |
| EuroQol ‘Today’ score, median (IQR) | 85 (75, 95) | 90 (80, 95) | 5 (-10, 15) | 0.079 |

Legend: IQR, interquartile range.

*Major health event – x1 arthroscopic release of psoas muscle; x 1gluteal muscle repair.
