# Supplementary material for: The effectiveness of inpatient rehabilitation after uncomplicated total hip arthroplasty: a propensity score matched cohort
Source: BMC Musculoskelet Disord. 2018 Jul 18;19:236. doi: 10.1186/s12891-018-2134-3 (PMC6052669; doi:10.1186/s12891-018-2134-3)
Supplement: Supplementary file 3 — Sensitivity analysis 2. Sensitivity analysis to determine effect of ‘hospital’ and ‘surgeon’. (DOCX 39 kb) [file 12891_2018_2134_MOESM3_ESM.docx]

**Additional file 3**

**The effectiveness of inpatient rehabilitation after uncomplicated total hip arthroplasty: a propensity score matched cohort**

**Naylor JM, Hart A, Mittal R, Harris IA, Xuan W**

**Sensitivity analysis to determine effect of ‘hospital’ and ‘surgeon’**

**Hospital**

Results of multiple linear regression analyses on the unmatched cohort to determine whether ‘hospital’ influenced the effect of treatment group. Three regression models were developed for each primary outcome at each point in time. Model 1 was the unadjusted effect of treatment. Model 2 included the effect of the propensity score for each person. Model 3 included ‘hospital’. Note that for almost all unadjusted comparisons excluding OHS 365-day, outcomes were worse in the Inpatient Rehabilitation group. The addition of the propensity score to the model (ie balancing the groups on their probability of receiving inpatient therapy based on the measured covariates), removed the effect of treatment on the outcome. In other words, inpatient rehabilitation was no longer associated with a worse outcome at almost all times points for both the EQVAS and OHS.

The significant influence of adding ‘hospital’ as a covariate is demonstrated if the association between treatment group and outcome varies between Model 2 and Model 3; in other words; the addition of ‘hospital’ changes the significance level of the treatment from significant to non-significant, or vice versa. For all the models, hospital did not have a statistically measurable effect.

**Day 35 EQVAS**

| Model | Variable | Beta coefficient | P-value |
| --- | --- | --- | --- |
| 1 | Inpatient Yes/No | -3.5 | 0.0006 |
| 2 | Inpatient Yes/No | -2.3 | 0.09 |
|  | Propensity score | -7.7 | 0.03 |
| 3 | Inpatient Yes/No | -2.5 | 0.07 |
|  | Propensity score | -7.4 | 0.04 |
|  | Hospital | Not applicable* | 0.34 |

*Hospital defined as categorical. There were too many hospital categories so determination of 1 simple co-efficient was not possible. This applies to all the models presented in Appendix 2.

Interpretation - ‘Hospital’ is not influencing the association between treatment (Inpatient Yes/No) and the outcome as there is no change to the p-value (significant to non-significant, or vice versa) for ‘Inpatient Yes/No’ between Model 2 and 3 (see shaded values).

**Day 90 EQVAS**

| Model | Variable | Beta coefficient | P-value |
| --- | --- | --- | --- |
| 1 | Inpatient Yes/No | -2.54 | 0.049 |
| 2 | Inpatient Yes/No | -1.13 | 0.44 |
|  | Propensity score | -7.2 | 0.06 |
| 3 | Inpatient Yes/No | -1.41 | 0.33 |
|  | Propensity score | -6.8 | 0.07 |
|  | Hospital | Not applicable | 0.11 |

Interpretation – ‘Hospital’ is not influencing the association between treatment (Inpatient Yes/No) and the outcome as there is no change to the p-value (significant to non-significant, or vice versa) for ‘Inpatient Yes/No’ between Model 2 and 3 (see shaded values).

**Day 365 EQVAS**

| Model | Variable | Beta coefficient | P-value |
| --- | --- | --- | --- |
| 1 | Inpatient Yes/No | -3.95 | 0.0027 |
| 2 | Inpatient Yes/No | -2.18 | 0.13 |
|  | Propensity score | -8.47 | 0.02 |
| 3 | Inpatient Yes/No | -2.32 | 0.11 |
|  | Propensity score | -8.27 | 0.028 |
|  | Hospital | Not applicable | 0.49 |

Interpretation – ‘Hospital’ is not influencing the association between treatment (Inpatient Yes/No) and the outcome as there is no change to the p-value (significant to non-significant, or vice versa) for ‘Inpatient Yes/No’ between Model 2 and 3 (see shaded values). .

**Day 90 Oxford Hip Score**

| Model | Variable | Beta coefficient | P-value |
| --- | --- | --- | --- |
| 1 | Inpatient Yes/No | -1.57 | 0.006 |
| 2 | Inpatient Yes/No | -0.89 | 0.16 |
|  | Propensity score | -3.88 | 0.0176 |
| 3 | Inpatient Yes/No | -0.87 | 0.17 |
|  | Propensity score | -3.91 | 0.0172 |
|  | Hospital | Not applicable | 0.79 |

Interpretation - ‘Hospital’ is not influencing the association between treatment (Inpatient Yes/No) and the outcome as there is no change to the p-value (significant to non-significant, or vice versa) for ‘Inpatient Yes/No’ between Model 2 and 3 (see shaded values).

**Day 365 Oxford Hip Score**

| Model | Variable | Beta coefficient | P-value |
| --- | --- | --- | --- |
| 1 | Inpatient Yes/No | -0.82 | 0.077 |
| 2 | Inpatient Yes/No | -0.28 | 0.59 |
|  | Propensity score | -2.86 | 0.03 |
| 3 | Inpatient Yes/No | -0.32 | 0.55 |
|  | Propensity score | -2.81 | 0.04 |
|  | Hospital | Not applicable | 0.56 |

Interpretation - ‘Hospital’ is not influencing the association between treatment (Inpatient Yes/No) and the outcome as there is no change to the p-value (significant to non-significant, or vice versa) for ‘Inpatient Yes/No’ between Model 2 and 3 (see shaded values).

**Surgeon effect**

As for ‘hospital’, we performed multiple linear regression analyses on the unmatched cohort to determine whether ‘surgeon’ influenced the effect of treatment group.

Three regression models were developed for each primary outcome at each point in time. Model 1 was the unadjusted effect of treatment. Model 2 included the effect of the propensity score for each person. Model 3 included ‘surgeon’. Surgeons who only referred patients to either inpatient rehabilitation all the time OR home all the time were excluded. This was necessary as each surgeon needed to exhibit some variation in referral destination to be included in the model. Hence, the patient sample reduced to n = 238. New propensity scores were calculated for the new reduced sample.

The significant influence of adding ‘surgeon’ as a covariate is demonstrated if the association between treatment group and outcome varies between Model 2 and Model 3; in other words; the addition of ‘surgeon’ changes the significance level of the treatment from significant to non-significant, or vice versa. For all the models, ‘surgeon’ did not have statistically measurable effect.

**Day 35 EQVAS**

| Model | Variable | Beta coefficient | P-value |
| --- | --- | --- | --- |
| 1 | Inpatient Yes/No | -3.60 | 0.02 |
| 2 | Inpatient Yes/No | -2.27 | 0.21 |
|  | Propensity score | -4.41 | 0.25 |
| 3 | Inpatient Yes/No | -2.52 | 0.16 |
|  | Propensity score | -4.03 | 0.29 |
|  | Surgeon | Not applicable* | 0.52 |

*Surgeon defined as categorical. There were too many categories so determination of 1 simple co-efficient was not possible. This applies to all the models presented in Appendix 2.

Interpretation - ‘Surgeon’ is not influencing the association between treatment (Inpatient Yes/No) and the outcome as there is no change to the p-value (significant to non-significant, or vice versa) for ‘Inpatient Yes/No’ between Model 2 and 3.

**Day 90 EQVAS**

| Model | Variable | Beta coefficient | P-value |
| --- | --- | --- | --- |
| 1 | Inpatient Yes/No | -2.88 | 0.08 |
| 2 | Inpatient Yes/No | -2.61 | 0.17 |
|  | Propensity score | 0.02 | 0.99 |
| 3 | Inpatient Yes/No | -2.32 | 0.22 |
|  | Propensity score | -0.17 | 0.97 |
|  | Surgeon | Not applicable | 0.82 |

Interpretation – ‘Surgeon’ is not influencing the association between treatment (Inpatient Yes/No) and the outcome as there is no change to the p-value (significant to non-significant, or vice versa) for ‘Inpatient Yes/No’ between Model 2 and 3.

**Day 365 EQVAS**

| Model | Variable | Beta coefficient | P-value |
| --- | --- | --- | --- |
| 1 | Inpatient Yes/No | -4.34 | 0.01 |
| 2 | Inpatient Yes/No | -2.58 | 0.18 |
|  | Propensity score | -6.07 | 0.13 |
| 3 | Inpatient Yes/No | -2.51 | 0.19 |
|  | Propensity score | -5.48 | 0.18 |
|  | Surgeon | Not applicable | 0.37 |

Interpretation – ‘Surgeon’ is not influencing the association between treatment (Inpatient Yes/No) and the outcome as there is no change to the p-value (significant to non-significant, or vice versa) for ‘Inpatient Yes/No’ between Model 2 and 3.

**Day 90 Oxford Hip Score**

| Model | Variable | Beta coefficient | P-value |
| --- | --- | --- | --- |
| 1 | Inpatient Yes/No | -0.97 | 0.16 |
| 2 | Inpatient Yes/No | -0.50 | 0.54 |
|  | Propensity score | -1.75 | 0.31 |
| 3 | Inpatient Yes/No | -0.35 | 0.65 |
|  | Propensity score | -1.44 | 0.38 |
|  | Surgeon | Not applicable | 0.06 |

Interpretation - ‘Surgeon’ is not influencing the association between treatment (Inpatient Yes/No) and the outcome as there is no change to the p-value (significant to non-significant, or vice versa) for ‘Inpatient Yes/No’ between Model 2 and 3.

**Day 365 Oxford Hip Score**

| Model | Variable | Beta coefficient | P-value |
| --- | --- | --- | --- |
| 1 | Inpatient Yes/No | -0.95 | 0.09 |
| 2 | Inpatient Yes/No | -0.56 | 0.38 |
|  | Propensity score | -1.54 | 0.26 |
| 3 | Inpatient Yes/No | -0.56 | 0.39 |
|  | Propensity score | -1.26 | 0.35 |
|  | Surgeon | Not applicable | 0.14 |

Interpretation - ‘Surgeon’ is not influencing the association between treatment (Inpatient Yes/No) and the outcome as there is no change to the p-value (significant to non-significant, or vice versa) for ‘Inpatient Yes/No’ between Model 2 and 3.
