# Supplementary material for: The effectiveness of inpatient rehabilitation after uncomplicated total hip arthroplasty: a propensity score matched cohort
Source: BMC Musculoskelet Disord. 2018 Jul 18;19:236. doi: 10.1186/s12891-018-2134-3 (PMC6052669; doi:10.1186/s12891-018-2134-3)
Supplement: Supplementary file 4 — Sensitivity analysis 3. Sensitivity analysis to determine effect of the unmeasured confounders. (DOCX 21 kb) [file 12891_2018_2134_MOESM4_ESM.docx]

**Additional file 4**

**The effectiveness of inpatient rehabilitation after uncomplicated total hip arthroplasty: a propensity score matched cohort**

**Naylor JM, Hart A, Mittal R, Harris IA, Xuan W**

**Sensitivity analysis to determine the possible effect of the unmeasured confounders**

Account for unmeasured confounders

|  | Gamma^*^ | P-value^#^  (un-Confounded) | Lower boundary^@^ | Upper boundary^@^ |
| --- | --- | --- | --- | --- |
| **1.Day 35 EQVAS** | 1.0 | 0.117 | 0.117 | 0.117 |
|  | 1.2 |  | 0.0224 | 0.3531 |
| **2.Day 90 EQVAS** | 1.0 | 0.2421 | 0.2421 | 0.2421 |
|  | 1.3 |  | 0.0287 | 0.6875 |
| **3.Day 90 OHS** | 1.0 | 0.2996 | 0.2996 | 0.2996 |
|  | 1.3 |  | 0.0454 | 0.7359 |
| **4.Day 365 EQVAS** | 1.0 | 0.0466 | 0.0466 | 0.0466 |
|  | 1.1 |  | 0.017 | 0.107 |
| **5.Day 365 OHS** | 1.0 | 0.5458 | 0.5458 | 0.5458 |
|  | 1.6 |  | 0.0438 | 0.9741 |
| **6. Total provider charges** | 1.0 | <0.0001 | <0.0001 | <0.0001 |
|  | 6.0 |  | <0.0001 | <0.0001 |
| **7. Total minus inpatient rehabilitation charges** | 1.0 | <0.0001 | <0.0001 | <0.0001 |
|  | 5.5 |  | <0.0001 | 0.0798 |

Notes:

* Gamma is the sensitivity parameter that measures the degree of departure from the random assignment to inpatient group (Gamma=2 indicated a patient were twice as likely to be assigned to inpatient versus to “home” due to the effect of the unmeasured confounders).

# P-value to compare inpatients versus “home”, assuming no effect of un-measured confounding (Gamma=1) on inpatient group allocation. This p-value is equivalent to the p-value from Wilcoxon Sign Rank test in PS matched sample.

@ Lower and Upper boundary indicates the range of the p-values with the corresponding minimized Gamma that produced both significant and insignificant results within the interval (except for ‘Total provider charges’, where a Gamma=6 still end up with a highly significant difference between inpatient versus home on that measurement).
